# Tumor Growth Ameliorates Cardiac Dysfunction and Suppresses Fibrosis in a Mouse Model for Duchenne Muscular Dystrophy

**DOI:** 10.3390/ijms241612595

**Published:** 2023-08-09

**Authors:** Laris Achlaug, Lama Awwad, Irina Langier Goncalves, Tomer Goldenberg, Ami Aronheim

**Affiliations:** Department of Cell Biology and Cancer Science, Ruth and Bruce Rappaport Faculty of Medicine, Technion—Israel Institute of Technology, P.O. Box 9649, Haifa 31096, Israel; laris1010.ab@gmail.com (L.A.); lamaaw@campus.technion.ac.il (L.A.); irinalan@campus.technion.ac.il (I.L.G.); tomergo@campus.technion.ac.il (T.G.)

**Keywords:** Duchenne Muscular Dystrophy, fibrosis, cardiac remodeling, cardiac dysfunction, tumor, macrophage recruitment

## Abstract

The interplay between heart failure and cancer represents a double-edged sword. Whereas cardiac remodeling promotes cancer progression, tumor growth suppresses cardiac hypertrophy and reduces fibrosis deposition. Whether these two opposing interactions are connected awaits to be determined. In addition, it is not known whether cancer affects solely the heart, or if other organs are affected as well. To explore the dual interaction between heart failure and cancer, we studied the human genetic disease Duchenne Muscular Dystrophy (DMD) using the MDX mouse model. We analyzed fibrosis and cardiac function as well as molecular parameters by multiple methods in the heart, diaphragm, lungs, skeletal muscles, and tumors derived from MDX and control mice. Surprisingly, cardiac dysfunction in MDX mice failed to promote murine cancer cell growth. In contrast, tumor-bearing MDX mice displayed reduced fibrosis in the heart and skeletal and diaphragm muscles, resulting in improved cardiac function. The latter is at least partially mediated via M2 macrophage recruitment to the heart and diaphragm muscles. Collectively, our data support the notion that the effect of heart failure on tumor promotion is independent of the improved cardiac function in tumor-bearing mice. Reduced fibrosis in tumor-bearing MDX mice stems from the suppression of new fibrosis synthesis and the removal of existing fibrosis. These findings offer potential therapeutic strategies for DMD patients, fibrotic diseases, and cardiac dysfunction.

## 1. Introduction

Fibrosis, the thickening or scarring of a tissue, is part of the wound-healing process [1,2]. Pathophysiological fibrosis is observed in several diseases, including ones involving the liver [3], heart [4,5], skeletal muscles [6], kidneys [7], and lungs [8]. Collectively, fibrotic diseases account for up to half of deaths in the developed world and are clearly an unmet clinical need [9]. We have previously shown that heart failure in tumor-bearing mice results in an altered gene expression program in the heart, tumor, and other organs [10,11]. These changes lead to the secretion of multiple factors that promote tumor growth and metastasis spread [10,11,12]. Yet, we have also found that tumor growth suppresses cardiac remodeling, reduces cardiac hypertrophy, and inhibits de novo synthesis of fibrosis in mice with cardiac dysfunction [13]. Whether the interplay between heart failure and tumor growth is connected to cardiac dysfunction amelioration in tumor-bearing mice is yet to be determined. It is also unknown whether the tumor-dependent effects on cardiac function are specific to the failing heart or represent a systemic effect. In order to investigate these inquiries within the context of a human disease with clinical relevance, we utilized the MDX mouse model for Duchenne Muscular Dystrophy (DMD). DMD is a muscular disorder that impacts approximately one in 3500 males, resulting from an X-linked loss-of-function mutation in the dystrophin gene. Individuals affected by DMD experience muscle fibrosis in skeletal, cardiac, and diaphragm muscles, as well as lung complications [14,15]. Additionally, DMD patients commonly exhibit cardiomyopathy and muscle pathologies. By subcutaneously implanting cancer cells into C57Bl/10 (control) and MDX mice, we were able to monitor tumor growth, fibrosis, and cardiac contractile function. Although MDX mice displayed clear cardiac dysfunction, no potentiation of tumor growth was observed. Nevertheless, tumor growth did, however, significantly suppress de novo synthesis of fibrosis deposition and significantly reduce existing fibrosis in the heart and skeletal and diaphragm muscles, resulting in improved cardiac contractile function. This occurred, at least in part, via macrophage M2 recruitment to the heart. These cancer paradigms may provide novel therapeutic strategies towards the treatment of human fibrotic diseases.

## 2. Results

### 2.1. Tumor Growth Significantly Improved the Contractile Function of MDX Heart and Diaphragm Muscles

**Figure 1:**
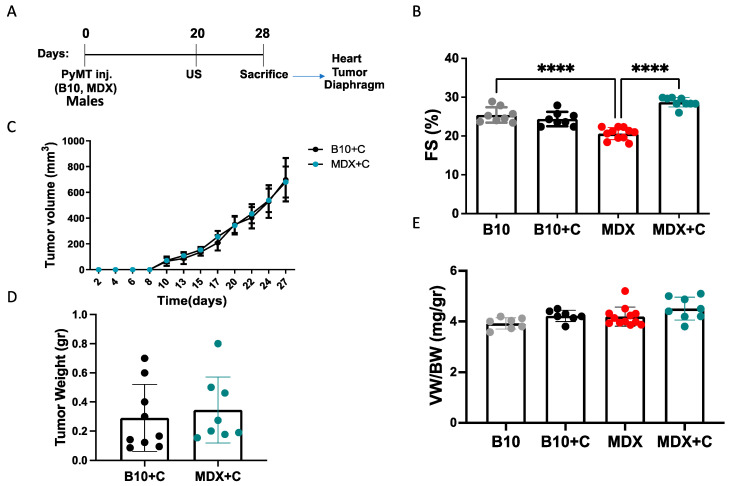
Cardiac dysfunction fails to promote tumor growth, but tumor growth is accompanied by improved cardiac contractile function. (**A**) Schematic representation of the experimental timeline. C57Bl/10 and MDX male mice (6 months old) were injected in the flanks with PyMT cells (10^6^ cells per mouse) or left untreated (control). Echocardiography (US) was performed 8 days prior to sacrifice. (**B**) The measured fractional shortening (FS) in C57Bl/10 (B10), MDX, and tumor-bearing mouse groups (B10+C and MDX+C). FS was assessed by echocardiography and calculated using the formula: FS (%) = [(LVDd- LVDs)/LVDd]. (**C**) Tumor volume (width^2^ × length × 0.5) was monitored over time in tumor-bearing C57Bl/10 and MDX mice (**D**) Tumor weight at the endpoint in C57Bl/10 and MDX mice cohorts. (**E**) Ventricular weight to body weight ratio (VW/BW). Data are presented as mean ± SE. One-way ANOVA followed by Tukey post-test (**B**,**E**); two-way ANOVA with Bonferroni repeated measure (**C**) or Student’s *t*-test (**D**). **** *p* < 0.0001. Each dot represents one mouse.

To examine how cardiac dysfunction affects tumor growth in the DMD mouse model, breast cancer PyMT cells were implanted into the flanks of six-month-old MDX male mice. As the MDX mouse strain is under a C57Bl/10 background, C57Bl/10 mice served as a disease-free control. Tumor growth was monitored over time, and cardiac function was assessed using echocardiography prior to sacrifice, after which fractional shortening (FS) was calculated (Figure 1A). MDX male mice displayed significantly lower contractile function than C57Bl/10 mice, with the former reaching a FS of 20% compared to 25% in the latter (Figure 1B and Supplemental Table S1). Nevertheless, tumor volume was similar in the two cohorts and throughout the experiment (Figure 1C), and the tumor weight was similar at the endpoint (Figure 1D). Interestingly, cardiac dysfunction was not accompanied by heart hypertrophy, as ventricular weight to body weight ratio (VW/BW) was similar in both C57Bl/10 and MDX mice (Figure 1E). Similar results were obtained when MDX and C57Bl/10 male mice were implanted with Lewis lung carcinoma cells (LLC) (Supplemental Figure S1A–D, Supplemental Table S2). These findings suggest that the cardiac dysfunction observed in MDX mice occurs independent of cardiac hypertrophy and is not sufficient to promote tumor growth in this model. In addition, though the FS in C57Bl/10 tumor-bearing mice is unchanged as compared with C57Bl/10 non-tumor-bearing, in the tumor-bearing MDX mice (PyMT and LLC), cardiac function was significantly improved (Figure 1B and Figure S1D).

**Figure 2:**
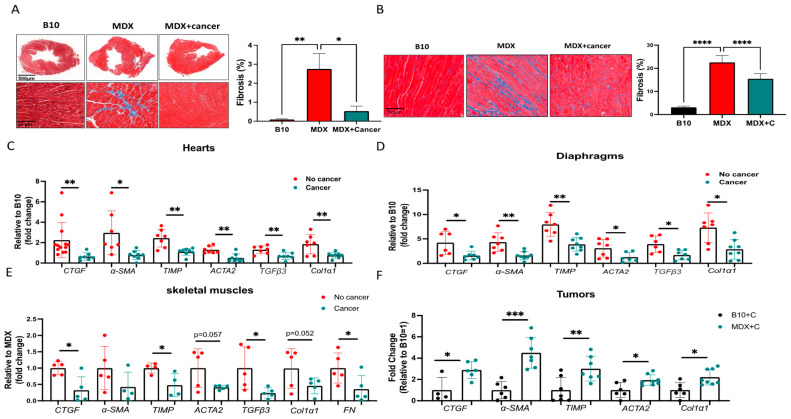
Tumor growth suppresses fibrosis hallmark gene markers transcription in MDX mice. (**A**,**B**) Left panel: Representative image of heart (**A**) and diaphragm (**B**) sections of B10, (left), MDX mouse (middle) and tumor-bearing MDX mouse (right) stained for Masson’s trichrome. Scale bar: 500 µm (top) and 50 µm (bottom). Right panel: Percent of interstitial fibrosis, quantified using ImageJ software, based on at least five fields from each individual mouse in each cohort (*n* = 5). (**C**–**E**) Transcription levels of fibrosis hallmark gene markers CTGF, αSMA, TIMP, ACTA2, TGFβ3, Col1α1, and FN (in skeletal muscles) in the heart (**C**), diaphragm (**D**), and skeletal muscles (**E**) of non-tumor-bearing and tumor-bearing MDX mice, measured using qRT-PCR normalized to Hsp90 (heart) mb2m (diaphragms and skeletal muscles). (**F**) Transcription levels of fibrosis hallmark gene markers CTGF, αSMA, TIMP, ACTA2, and Col1α1 in the tumors of C57Bl/10 and MDX mice, measured using qRT-PCR normalized to β-actin. Data are presented as the relative expression compared to C57/Bl/10 mice (determined as 1) in the hearts, diaphragms, and tumors and compared to MDX (determined as 1) in skeletal muscles. Results are presented as mean ± SE; one-way repeated measures ANOVA followed by Tukey posttests (**A**–**D**) or Student’s *t*-test (**E**,**F**). * *p* < 0.05; ** *p* < 0.01; *** *p* < 0.001, **** *p* < 0.0001. Each dot represents one mouse.

Reduced cardiac contractile function in the MDX mouse model is typically associated with fibrosis in the heart. Therefore, we examined the extent of fibrosis in heart sections derived from C57Bl/10 (B10), PyMT tumor-bearing, and non-tumor-bearing MDX mice by staining them with Masson’s Trichrome. Indeed, tumor-bearing MDX mice displayed much lower fibrosis staining compared to aged-matched MDX mice with no tumor (Figure 2A). Diaphragms of tumor-bearing MDX mice were also stained with Masson’s Trichome in order to evaluate the extent of fibrosis. Similar to the heart, diaphragms of tumor-bearing MDX mice displayed much lower fibrosis staining compared to aged-matched MDX mice with no tumor (Figure 2B). These reductions were accompanied by reduced levels of fibrosis hallmark gene markers, as evaluated by qRT-PCR using mRNA derived from the heart, diaphragm muscles, and skeletal muscles (Figure 2C–E). Interestingly, tumors derived from MDX mice exhibited elevated transcription levels of fibrosis hallmark gene markers compared to tumors of a similar size derived from C57Bl/10 mice (Figure 2F). These results suggest that tumors display increased fibrosis gene programming in MDX mice compared to C57Bl/10 mice, and the heart, diaphragm muscles, and skeletal muscles exhibit reduced fibrosis.

Similar experiments were repeated in an orthotopic cancer model. Namely, PyMT cells were implanted into the mammary fat pad of MDX and C57Bl/10 female mice (Supplemental Figure S2A). Consistent with the MDX male mice, tumor-bearing MDX female mice exhibited improved cardiac contractile function, as observed by elevated FS (Supplemental Figure S2B, Supplemental Table S3) and a reduction in fibrosis hallmark gene markers in heart and diaphragm muscles following tumor implantation, as assessed by qRT-PCR (Supplemental Figure S2C,D). In addition, the lung weight to body weight ratio was lower in MDX female mice as compared with C57Bl/10 female mice. The LW/BW ratio was elevated in tumor-bearing female MDX mice (Supplemental Figure S3A). This increase in lung weight in tumor-bearing mice was accompanied by a reduction in fibrosis hallmark gene markers compared to the MDX female mouse cohort (Supplemental Figure S3B).

### 2.2. Improved Contractile Function and Reduction in Fibrosis in Tumor-Bearing Mice Is Mediated by M2 Macrophage Recruitment to the Heart

**Figure 3:**
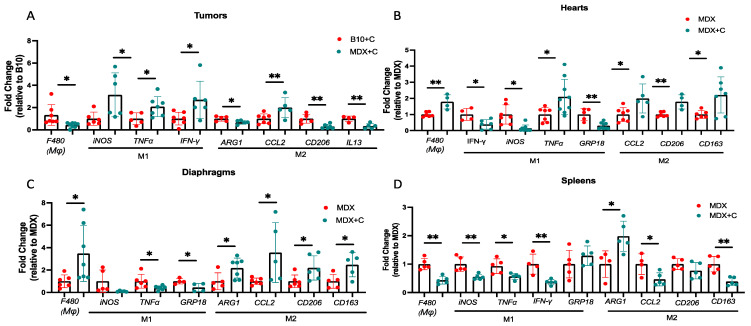
Tumor growth in MDX mice induces the expression of M2-polarizing hallmark gene markers. (**A**–**D**) qRT-PCR measuring transcription levels of macrophage hallmark gene markers in the tumors as compared with C57Bl/10 (**A**) in the hearts (**B**), diaphragm muscles (**C**), and spleens (**D**). Gene expression compares non-tumor-bearing and PyMT-tumor-bearing MDX male mice (same experimental cohort as in Figure 1). Measurements were obtained using qRT-PCR, normalized to housekeeping gene Hsp90. Results are presented as mean ± SE, Student’s *t*-test. * *p* < 0.05; ** *p* < 0.01. Each dot represents one mouse.

A previous study suggested that innate immune cells are involved in tumor-dependent cardiac dysfunction amelioration [13]. Therefore, we sought to examine their role in global fibrosis reduction in the MDX mice model. Towards this end, we used qRT-PCR analysis of mRNA derived from PyMT tumors, hearts, diaphragms, and spleens of the male mice cohorts. We first compared the level of F4/80, a gene marker representing the total macrophage population, in the tumors derived from C57Bl/10 and MDX mice (Figure 3A). In general, we observed reduced F4/80 expression levels in tumors derived from MDX mice as compared with C57Bl/10 tumors. We then compared F4/80 expression levels in tissues derived from MDX mice in the absence and presence of tumors. F4/80 expression levels were higher in the heart and the diaphragm muscles of PyMT tumor-bearing MDX mice compared to non-tumor-bearing mice (Figure 3B,C). In contrast, F4/80 expression levels were lower in the spleens of tumor-bearing MDX mice (Figure 3D).

Macrophages are largely divided into two main functionally distinct forms, pro-inflammatory (M1) and anti-inflammatory (M2) [16,17]. To distinguish between the two macrophage populations, we examined the expression levels of specific M1 (iNOS, TNFα, IFN-γ) and M2 macrophage gene markers (ARG-1, CD206, CCL2, and IL-13) [16,17]. In tumors derived from MDX mice, we observed higher levels of M1 gene markers, whereas there were lower levels in the M2 gene markers as compared with tumors derived from C57Bl/10 (Figure 3A). We then compared the levels of M1-M2 hallmark gene markers in various tissues derived from tumor-bearing and non-tumor-bearing MDX mice. In the heart and diaphragm muscles of tumor-bearing MDX mice, we observed higher levels of M2 gene markers and lower levels of M1 gene markers (Figure 3B,C). In contrast, we observed a significant reduction in M1 gene markers in the spleens of tumor-bearing mice, whereas inconsistent changes were observed for the M2 markers (higher levels of Arg1 and lower levels of CCL2 and CD163) (Figure 3D). These results suggest that M1-to-M2 polarization occurs in the hearts and diaphragms of tumor-bearing MDX mice compared with MDX mice. This polarization is only partially observed in the spleens of tumor-bearing MDX mice.

**Figure 4:**
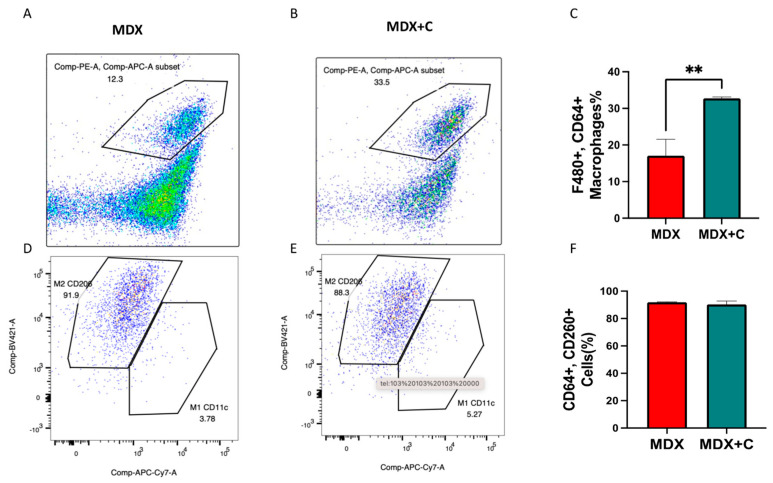
Tumor growth in MDX mice induces significant macrophage recruitment. (**A**,**B**) FACS analysis of total macrophage population in the hearts of naïve (n = 3) (**A**) and tumor-bearing (n = 5) MDX mice (**B**). These data are shown as the macrophages (%) F480+, CD64+ in (**C**). Additional analysis of M2 macrophage population CD64+CD206 in the hearts (**D**,**E**) and the quantification of % macrophages in each group (**F**). Results are presented as mean ± SEM, Student’s *t*-test. ** *p* < 0.01.

Macrophage recruitment was further supported by fluorescence-activated cell sorting (FACS) of hearts of non-tumor-bearing vs. tumor-bearing MDX mice. In the hearts, a significant elevation in the macrophage population (F480+, CD64+) was observed in tumor-bearing MDX mice compared with non-tumor-bearing mice (Figure 4A,B), even though M2 macrophage polarization occurred already in naive MDX mice, and no further elevation in M2 macrophage population occurred in tumor-bearing MDX mice as shown by RT-PCR using M1/M2 markers (Figure 4D–F, Supplemental Figure S4A,B). Nonetheless, the percent (%) of macrophages in the hearts of tumor-bearing mice was significantly elevated compared with the hearts of naïve MDX mice. Collectively, these results support the systemic alteration in the macrophage cell population towards M2 polarization, which is prominent in the heart.

Macrophage recruitment was further supported by fluorescence-activated cell sorting (FACS) of hearts of non-tumor-bearing vs. tumor-bearing MDX mice. In the hearts, a significant elevation in the macrophage population (F480+, CD64+) was observed in tumor-bearing MDX mice compared with non-tumor-bearing mice (Figure 4A,B), even though M2 macrophage recruitment occurred already in naive MDX mice, and the total macrophage population was significantly increased. Collectively, these results support the systemic alteration in the macrophage cell population towards M2 recruitment, which is prominent in the heart.

**Figure 5:**
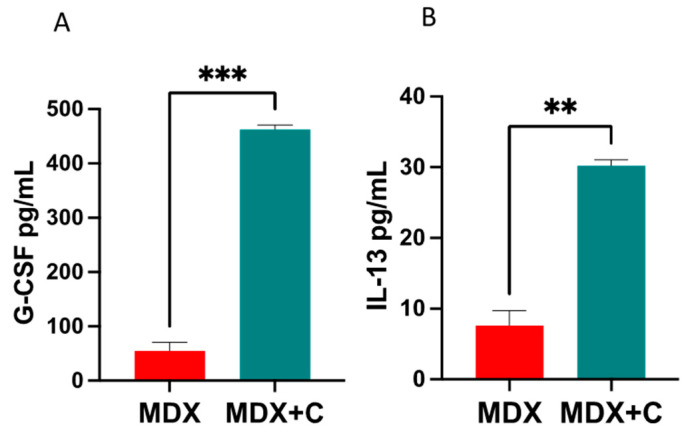
M2 polarizing cytokines G-CSF and IL-13 are elevated in the serum of PyMT-bearing compared with non-tumor-bearing MDX mice. Serum levels as in (**A**), obtained by ELISA for G-CSF and (**B**) IL-13. Pooled blood serum of MDX (n = 5 each) and PyMT-bearing MDX mice (n = 5) was used. Results are presented as mean ± SE, Student’s *t*-test. ** *p* < 0.01, *** *p* < 0.001.

We next examined two master regulatory factors associated with M1-M2 macrophages’ switch, G-CSF [18] and IL-13 [19] in the serum derived from MDX mice and tumor-bearing MDX mice. Significantly higher levels of both G-CSF and IL-13 were observed in the serum of tumor-bearing MDX mice as compared to MDX mice (Figure 5). This observation implies that these secreted factors could potentially play a pivotal role in promoting M2 macrophage recruitment to the heart.

**Figure 6:**
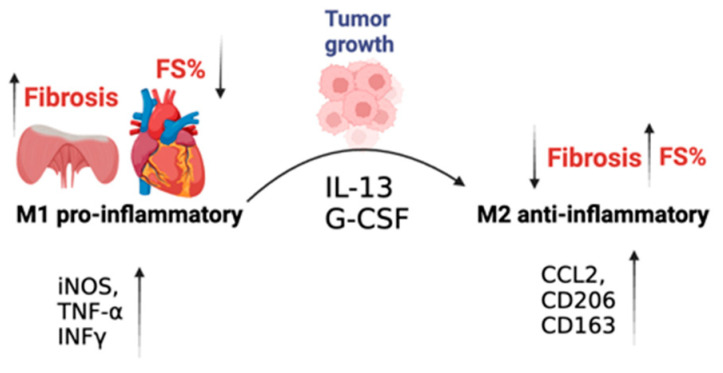
Graphical abstract describing the manuscript’s main findings. Tumor growth reduces fibrosis in the heart and diaphragm muscles and ameliorates cardiac contractile function. This occurs at least partially via M2 macrophage recruitment.

Collectively, our findings indicate that the MDX mice display reduced cardiac contractile function and massive fibrosis. Surprisingly, tumor-bearing MDX mice feature M2 macrophage recruitment to the heart. M2 macrophages are associated with significant muscle repair functions, as demonstrated by reduced matrix deposition and a reduction in the presence of fibrosis, which leads to the amelioration of cardiac contractile function and an overall beneficial phenotype. A schematic summary of the main manuscript findings and conclusions is provided in Figure 6.

## 3. Discussion

Fibrosis is an unmet clinical need in various diseases involving skeletal and cardiac muscles, lungs, liver, and kidneys [9]. One such disease is DMD, an X-linked genetic disease that involves cardiac, diaphragm, and skeletal muscle fibrosis [14]. Whereas previous studies have demonstrated that heart failure [10,11,20,21] promotes cancer progression, we found no promotion of cancer growth in the MDX mouse model for DMD, despite the fact that MDX mice demonstrate apparent cardiac dysfunction (FS 20%). Yet the tumor promotion phenotype was identified in multiple other mice models, such as those reflecting myocardial infarction (MI) [20,21,22], pressure overload (TAC) [10], and heart hypertrophy (ATF3 transgene) [11]. In addition, a chronic hypertension mouse model with heart hypertrophy lacking cardiac dysfunction resulted in enhanced tumor growth [12]. The MDX mouse model displayed reduced cardiac contractile function in the absence of cardiac hypertrophy. Therefore, we can conclude that the tumor promotion phenotype is dependent on cardiac hypertrophy and not a direct result of cardiac dysfunction.

To study the effect of tumor growth on cardiac function and fibrosis in the MDX mouse model, we analyzed the heart contractile function of MDX mice in the presence and absence of tumors. Cardiac contractile function was improved in tumor-bearing MDX mice and was comparable to the levels found in naïve C57Bl/10 mice. Naïve C57Bl/10 mice displayed reduced basal cardiac contractile function as compared to naïve C57Bl/6 tumor growth. Nevertheless, in tumor-bearing C57Bl/10, no improvement of cardiac contractile function was observed as compared with non-tumor-bearing C57Bl/10 mice.

The transcription of fibrosis hallmark gene markers in the lungs, heart, and skeletal and diaphragm muscles of MDX mice was suppressed significantly, the fibrosis staining revealed a considerable reduction in fibrosis both in the heart and diaphragm. This finding strongly indicates that tumor growth not only inhibited the development of new fibrosis hallmark gene markers but also promoted the dissolution of existing fibrotic scars. Moreover, the observation of decreased expression of fibrosis hallmark gene markers in the skeletal and diaphragm muscles and lungs suggests that the suppression of fibrosis due to tumor growth is not limited to specific tissues but is rather a widespread phenomenon. These results may have broader implications and relevance for other fibrotic disease models, suggesting potential systemic effects of tumor-induced fibrosis suppression.

Our findings indicate that these beneficial tumor effects on fibrosis are mediated via macrophage recruitment. In the tumor, the M1 macrophage population is increased, whereas in the heart and diaphragm muscles, there was an increase mostly in the total M2 macrophage population. This was demonstrated by qRT-PCR, FACS analysis, and ELISA using M2-polarizing secreted factors in the serum. Specifically, in the heart and diaphragm muscles, the local synthesis of the M2-polarizing cytokine CCL2 was elevated. Notably, CCL2 has been shown to play an important role in the prevention of LV dysfunction and remodeling after MI [23]. Similarly, M2 macrophages are thought to accelerate cardiac and tissue repair processes [17,24,25]. In contrast, in the tumor, the M1-polarizing cytokines iNOS, TNF-α, and INFγ are prominent [17]. Importantly, M2 macrophages’ contribution to reducing fibrosis in various organs strongly indicates their therapeutic side effect under the scenario of a growing tumor. The noted reduction in the spleen in M1 hallmark gene markers and the elevation in M2 gene markers may indicate that spleen-derived macrophages initiate the polarization of classically activated (M1) to alternatively activated (M2) macrophages. The final macrophage polarization occurs upon their recruitment to the fibrotic site, such as in the lungs, heart, and diaphragm muscles [26]. The recruitment of the F480^+^/CD64^+^ cell population in the heart in tumor-bearing MDX mice was further supported by FACS analysis. Furthermore, the M2-polarizing factors G-CSF and IL-13 were shown by ELISA to be elevated in tumor-bearing MDX mice as compared with non-tumor-bearing MDX mice. A previous study showed that IL-13 induces M2 polarization, leading to improved cardiac function and reduced heart injury in a viral myocarditis mouse model [19]. G-CSF was shown to induce macrophage polarization and mobilization in previous studies [27]. Furthermore, G-CSF has been shown to decrease inflammatory processes and to act positively on the process of peripheral nerve regeneration during the course of muscular dystrophy [18]. It was shown that already in four-week-old MDX mice, the M2 macrophage population dominates skeletal muscles [28]. Nonetheless, the M2 population supports fibrosis, whereas the increase in the M2 population in tumor-bearing MDX mice appears to be associated with repair and improvement in cardiac function.

In summary, in the MDX mouse model for DMD, cardiac dysfunction in the absence of heart hypertrophy fails to promote tumor growth. On the other hand, tumor growth in MDX mice reduces fibrosis in the lungs, heart, and skeletal and diaphragm muscles. The tumor paradigms identified here could serve as novel therapeutic strategies for the treatment of the devastating DMD disease. They may also have a beneficial systemic outcome for other fibrotic diseases.

Limitations: The study is limited to concluding the effect of cancer growth on cardiac fibrosis and function in MDX mice. In DMD human patients, fibrosis treatment is limited, and cancer is not considered an optional treatment. The identification of factors that are responsible for the beneficial effects here may be used as a potential therapeutic treatment.

Clinical perspectives: The manuscript describes, for the first time, a beneficial effect of cancer on cardiac dysfunction and fibrosis in a clinically relevant mouse model. Tumor growth ameliorates cardiac dysfunction and reduces overall fibrosis. Fibrosis diseases account for more than half deaths worldwide and represent an unmet need. Harnessing tumor paradigms may serve as a novel strategy to improve cardiac function and fibrosis diseases.

## 4. Materials and Methods

### 4.1. Animals

The C57BL/10ScSn-Dmd (MDX) and C57BL/10J (B10) mice were acquired from the Jackson Laboratory. They were bred and raised from birth at the Pre-Clinical Research Authority, located at the Ruth and Bruce Rappaport Faculty of Medicine, Technion. The mice were kept in mating cages with regular access to food and water. At three weeks of age, they were weaned into individual cages.

### 4.2. Cell Culture

Polyoma Middle T (PyMT) murine breast carcinoma cells were derived from primary tumor-bearing transgenic mice expressing PyMT under the control of the murine mammary tumor virus promoter [29]. The PyMT cells were kindly provided by Prof. Tsonwin Hai (Ohio State University, Columbus, OH, USA). The LLC cancer cell line was purchased from the American Type Culture Collection ATCC. Both cell lines were tested by IDEXX BioAnalytics and found to be free of *Mycoplasma* and viral contaminations. Cells were cultured in DMEM containing 10% FBS, 1% streptomycin and penicillin, 1% l-glutamine, and 1% sodium pyruvate at 37 °C in a humidified atmosphere containing 5% CO_2_. Cancer cell implantation was conducted at maximal passage number five.

### 4.3. Cancer Cell Implantation

PyMT cancer cells and LLC cancer cells were injected into the mammary fat pad of female mice (10^6^ cells per mouse) and into the back flanks of mice. The tumor size was measured using a caliper, and the tumor volume was calculated using the formula: Width^2^ × Length × 0.5. The humane endpoint is considered to be when a tumor reaches a size of 1500 mm^3^, according to the Institutional Animal Care and Use Committee.

### 4.4. Echocardiography

Mice were anesthetized with 1% isoflurane and kept on a 37 °C heated plate throughout the procedure. Echocardiography was performed with a Vevo3100 micro-ultrasound imaging system (VisualSonics, Fujifilm, Tokyo, Japan) equipped with 13–38 MHz (MS 400) and 22–55 MHz (MS550D) linear array transducers. Cardiac size, shape, and function were analyzed using conventional two-dimensional imaging and M-mode recordings. Maximal left ventricular end-diastolic (LVDd) and end-systolic (LVDs) dimensions were measured in short-axis M-mode images. Fractional shortening (FS) was calculated with the following formula: FS% = [(LVDd − LVDs)/LVIDd] × 100. FS values are based on the average of at least three measurements for each mouse.

### 4.5. RNA Extraction

RNA was extracted from lungs, hearts, diaphragms, and tumors using an Aurum total RNA fatty or fibrous tissue kit (no. 732–6830, Bio-Rad, Hercules, CA, USA), according to the manufacturer’s instructions. cDNA was synthesized from 1000 ng purified mRNA with an iScript cDNA Synthesis Kit (no. 170–8891, Bio-Rad), according to the manufacturer’s instructions.

### 4.6. Quantitative Real-Time PCR

Quantitative real-time polymerase chain reaction (qRT-PCR) was performed with a QuantStudio3 (Thermofisher Scientific, 5823 Newton drive, Carlsbad, CA 92008, USA). Serial dilutions of a standard sample were included for each gene to generate a standard curve. Values were normalized to Hsp90, β-actin, and mb2M expression levels for the heart, diaphragm, tumor tissue, and lungs, respectively. All the oligonucleotide sequences that were used are listed in Supplementary Table S4.

### 4.7. Fibrosis Staining

Heart tissues and diaphragms were fixed in 4% formaldehyde overnight, embedded in paraffin, serially sectioned at 10 µm intervals, and then mounted on slides. Masson trichrome staining was performed according to the standard protocol. Images were acquired using a 3DHistech Pannoramic 250 Flash III (3DHISTECH Ltd, H-1141 Budapest, Öv u. 3., Hungary). Each section was fully scanned. The percent of interstitial fibrosis was determined as the ratio of the fibrosis area to the total area of the section using Image Pro Plus software. Each dot represents the mean of the values taken from at least five fields, derived from a single mouse.

### 4.8. Heart Single-Cell Suspension and Flow Cytometry

Heart single-cell suspension and flow cytometry was prepared as previously described [30]. Briefly, hearts were perfused, extracted, finely minced, and then incubated with digestive enzymes at 37 °C on a rocking shaker at 50 rpm for 45–60 min. Samples were homogenized with a 40 μm cell strainer. Red blood cells were lysed using ammonium-chloride-potassium (ACK) lysis buffer. Next, samples were centrifuged at 400× *g* for 5 min at 4 °C, and the pellet was then suspended with FACS buffer. Cells were immune-stained with the following anti-mouse antibodies: CD45-Alexa Fluor^®®^ 700 (BioLegend, 103128, San Diego, CA, USA), CD11b-PerCP (BioLegend, 101228, CA, USA), F480-PE (BioLegend, 123110, CA, USA), CD206-BV421 (BioLegend, 141717, CA, USA), CD64-APC (BioLegend, 161006, CA, USA), Ly-6G- Brilliant Violet 510™ (BioLegend, 127633, CA, USA), Ly-6C-PE/Cyanine7 (BioLegend, 128018, CA, USA), Propidium iodide (Sigma, 25535-16-4, Darmstadt, Germany). Cells were incubated (30 min, 4 °C) with the antibody mixture in a staining buffer (PBS containing 1% bovine serum albumin and 0.05% sodium azide) and then washed twice with a staining buffer. Cells were acquired using a LSRFortessa flow cytometer (BD Biosciences, Franklin Lakes, NJ, USA). The data were analyzed using FlowJo V.10 software (FlowJo, Ashland, OR, USA).

### 4.9. ELISA

ELISA quantification of candidate secreted factors’ protein levels in the blood was performed with a granulocyte-colony stimulating factor (G-CSF) mouse ELISA Kit (MCS00, R&D Systems, Minneapolis, MN, USA) and a Mouse IL-13 ELISA Kit (M1300CB, R&D systems), according to the manufacturer’s instructions.

### 4.10. Statistical Analysis

The data are presented as mean ± standard error (SE). All mice were included in each statistical analysis, unless they were euthanized for humane reasons before reaching the experimental endpoint. During data collection, the experimental groups were blinded to the researchers. The mice for each group were selected randomly. Each experimental group consisted of at least n = 5 mice.

To determine the statistical significance of tumor volume, a two-way repeated-measures ANOVA followed by the Bonferroni posttest was used. For comparisons between several means, a one-way ANOVA followed by the Tukey posttest was performed. For comparisons between two means, either a two-tailed Student’s *t*-test or Mann–Whitney U test was utilized.

All statistical analyses were conducted using GraphPad Prism 10 software. A significance level of *p* < 0.05 was considered statistically significant.

## Data Availability

All the obtained data used to support the findings of this study are available from the corresponding author upon reasonable request.

## Supplementary Materials

The following supporting information can be downloaded at: https://www.mdpi.com/article/10.3390/ijms241612595/s1.
