# Supplementary material for: Tumor Growth Ameliorates Cardiac Dysfunction and Suppresses Fibrosis in a Mouse Model for Duchenne Muscular Dystrophy
Source: Int J Mol Sci. 2023 Aug 9;24(16):12595. doi: 10.3390/ijms241612595 (PMC10454371; doi:10.3390/ijms241612595)
Supplement: Supplementary file 1 [file ijms-24-12595-s001.zip › ijms-2525283-supplementary.pdf]

## Supplemental Figures and legends

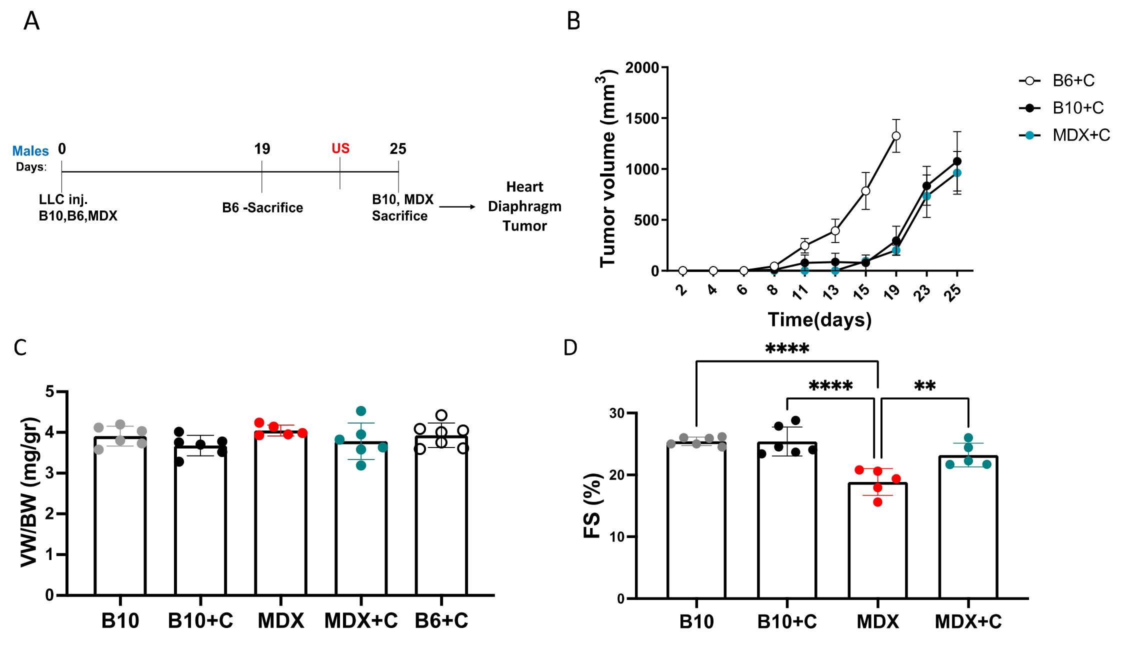


**Supplemental Figure S1. LLC cell implantation improves cardiac contractile function in MDX male mice.**

(A) Schematic experimental timeline. C57Bl/10, C57Bl/6 and MDX male mice were injected in the flanks with Lewis Lung carcinoma (LLC) cells ($0.5X10^6$ cells per mouse) or left untreated (control). Echocardiography (US) was performed prior to sacrifice (B) Tumor volume ($width^2$ X length X 0.5) over time in the LLC-tumor bearing C57Bl/10, C57Bl/6 and MDX mouse cohorts. (C) Ventricular weight to body weight ratio (VW/BW) at the endpoint in all cohorts. Each dot represents one mouse. (D) The calculated fractional shortening (FS) in C57Bl10 and MDX mice and LLC-tumor-bearing C57Bl/10 (B10 +C) and MDX (MDX + C) mice one day before endpoint. FS was assessed using echocardiography and calculated using the formula: FS (%) = [(LVDd - LVDs) / LVDd]. Data is represented as mean ± SE; one-way ANOVA followed by Turkey's multiple comparisons. **P<0.01, ****P<0.0001.

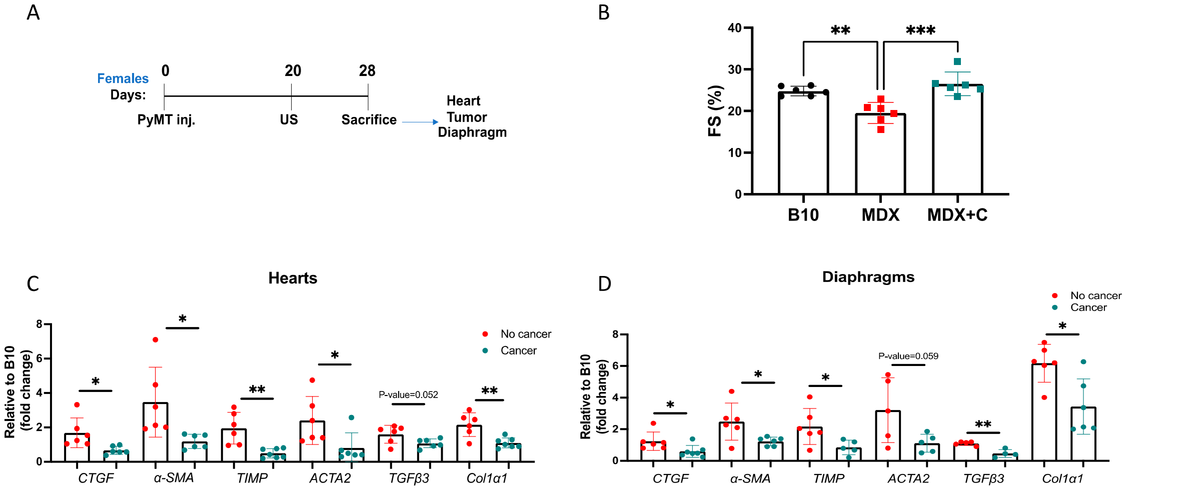


**Supplemental Figure S2. PyMT tumor growth improves cardiac function and suppresses fibrosis hallmark gene markers' transcription in heart and diaphragm muscles of MDX mice.**

(A) Schematic experimental timeline. MDX female mice were left untreated or were subcutaneously injected in the mammary fat pad with PyMT breast cancer cells ($10^6$ cells). C57Bl/10 mice served as control. Echocardiography was performed prior to sacrifice (US) (B) The measured fractional shortening (FS) in the C57Bl10, MDX and PyMT-tumor-bearing MDX female mice. FS was assessed using echocardiography and calculated using the formula: FS (%) = [(LVDd - LVDs) / LVDd]. (C-D) The relative transcription level of fibrosis hallmark gene markers of CTGF, αSMA, TIMP, ACTA2, TGFβ3 and Col1α1 in the heart (C) and diaphragm muscles (D) of MDX female mice in the absence and presence of PyMT-tumor-bearing measured using qRT-PCR. Results were normalized by housekeeping genes Hsp90, in the heart, and mb2M, in the diaphragm, and are presented as mean ± SEM; one-way ANOVA followed by Tukey post-test. $^*P<0.05$, $^{**}P<0.01$. Each dot represents one mouse.

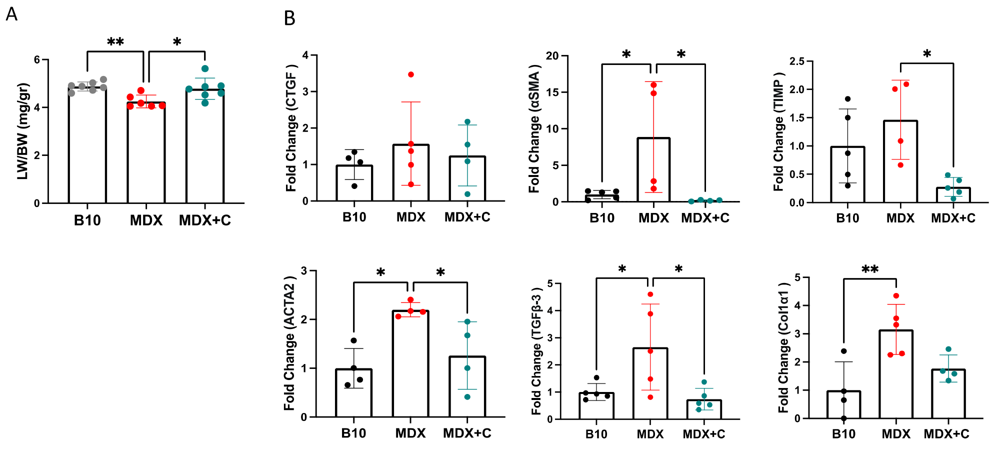


**Supplemental Figure S3. Tumor growth suppresses fibrosis hallmark gene markers' transcription** in the lungs of MDX male mice. (A) The lung's weight to body weight (LW/BW) ratio at the endpoint. (B). The transcription level of fibrosis hallmark gene markers CTGF, αSMA, TIMP, ACTA2, TGFβ3 and Col1α1 in the lungs derived from naïve C57Bl/10, naïve MDX and tumor-bearing MDX male mice, using qRT-PCR and normalized with Hsp90. Data are presented as the relative expression compared to naïve C57/Bl/10 mice (determined as 1). Results are presented as mean ± SE; one-way repeated measures ANOVA followed by Tukey posttests. *P<0.05; **P<0.01. Each dot represents one mouse.

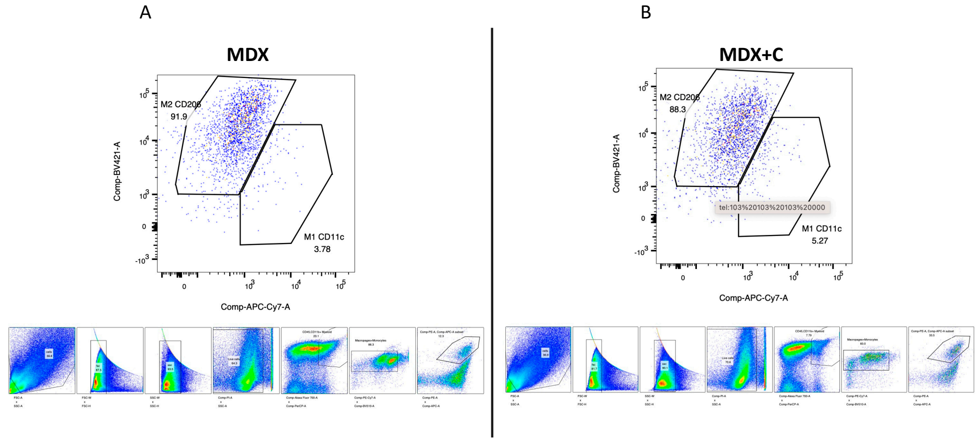


**Supplemental Figure S4. Tumor growth in MDX mice induces macrophage recruitment**. (A-B) FACS analysis and gating of hearts of naïve (n=3) (A) and tumor-bearing (n=5) MDX (B) mice.

| | B10 | B10+C | MDX | MDX+C |
|---|---|---|---|---|
| IVS;d-D | 0.72±0.13 | 0.76±0.16 | 0.93±0.13 | 0.72±0.12 |
| IVS;d-D | 1.06±0.15 | 1.10±0.17 | 1.26±0.17 | 1.08±0.12 |
| LVID;d-D | 3.73±0.21 | 3.83±0.27 | 3.94±0.19 | 3.6±0.14 |
| LVID;s-D | 2.78±0.18 | 2.89±0.2 | 3.12±0.17 | 2.52±0.13 |
| LVPW;d-D | 0.59±0.05 | 0.62±0.09 | 0.71±0.1 | 0.55±0.01 |
| LVPW;s-D | 0.90±0.07 | 0.92±0.09 | 0.84±0.14 | 0.89±0.09 |
| EF | 50.89±3.2 | 49.08±3.32 | 42.82±2.91 | 56.95±1.81 |
| Heart rate | 446±55.6 | 410±47.49 | 426.7±30.72 | 472.6±27.20 |
| FS | 25.43±1.99 | 24.35±2.03 | 20.58±1.63 | 28.72±1.24 |

**Supplemental Table S1. Echocardiography parameters of Fig.1B**

| | B10 | B10+C | MDX | MDX+C |
|---|---|---|---|---|
| IVS;d-D | 0.69±0.1 | 0.68±0.12 | 0.101±0.15 | 0.72±0.07 |
| IVS;d-D | 1.042±0.09 | 0.99±0.13 | 1.34±0.23 | 1.08±0.12 |
| LVID;d-D | 3.969±0.41 | 3.67±0.15 | 3.89±0.15 | 3.98±0.4 |
| LVID;s-D | 2.08±0.25 | 2.73±0.17 | 3.13±0.14 | 2.92±0.3 |
| LVPW;d-D | 0.62±0.05 | 0.57±0.05 | 0.73±0.1 | 0.59±0.04 |
| LVPW;s-D | 0.89±0.03 | 0.88±0.04 | 0.89±0.15 | 0.88±0.08 |
| EF | 49.86±2.09 | 51.15±4.17 | 41.4±4.33 | 52.49±3.65 |
| Heart rate | 430±36.16 | 451.2±56.86 | 407±22.32 | 409.83±24.27 |
| FS | 25.79±1.14 | 25.47±2.57 | 18.5±2.5 | 23.53±2.85 |

**Supplemental Table S2. Echocardiography parameters of Sup Fig.1D**

| | B10 | MDX | MDX+C |
|---|---|---|---|
| IVS;d-D | 0.67±0.08 | 1.01±0.15 | 0.74±0.06 |
| IVS;d-D | 1.02±0.07 | 1.34±0.23 | 1.11±0.12 |
| LVID;d-D | 3.86±0.3 | 3.89±0.15 | 4.09±0.45 |
| LVID;s-D | 2.9±0.3 | 3.13±0.14 | 3±0.33 |
| LVPW;d-D | 0.61±0.05 | 0.73±0.1 | 0.6±0.04 |
| LVPW;s-D | 0.87±0.05 | 0.89±0.15 | 0.9±0.06 |
| EF | 4994±2.02 | 41.14±4.33 | 52.41±3.65 |
| Heart rate | 431±32.71 | 407±22.32 | 407±22.36 |
| FS | 24.79±1.14 | 19.5±2.51 | 26.53±2.85 |

**Supplemental Table S3. Echocardiography parameters of Sup Fig.2B**

| Gene | Forward | Reversed |
|---|---|---|
| Hsp90 | TCGTCAGAGCTGATGATGAAGT | GCGTTTAACCCATCCAACTGAAT |
| mb2M | TTCTGGTGCTTGTCTCACTGA | CAGTATGTTCGGCTTCCCATTC |
| β-actin | GGCTGTATTCCCCTCCATCG | CCAGTTGGTAACAATGCCATGT |
| ACTA2 | GTCCCAGACATCAGGGAGTAA | TCGGATACTTCAGCGTCAGGA |
| Col1α1 | CTGGCGGTTCAGGTCCAAT | TTCCAGGCAATCCACGAGC |
| TGFβ3 | CCTGGCCCTGCTGAACTTG | GACGTGGGTCATCACCGAT |
| CTGF | AGACCTGTGGGATGGGCAT | GCTTGGCGATTTTAGGTGTCC |
| TIMP | GCAACTCGGACCTGGTCATAA | CGGCCCGTGATGAGAAACT |
| α-SMA | GTCCCAGACATCAGGGAGTAA | TCGGATACTTCAGCGTCAGGA |
| F4/80 | CCCCAGTGTCCTTACAGAGTG | GTGCCCAGAGTGGATGTCT |
| iNOS | GACATTACGACCCCTCCCAC | GCACATGCAAGGAAGGGAAC |
| CD206 | CTAACTGGGGTGCTGACGAG | GGCAGTTGAGGAGGTTCAGT |
| Arg1 | AATGAAGAGCTGGCTGGTGT | CTGGTTGTCAGGGGAGTGTT |
| CD163 | CCTCCTCATTGTCTTCCTCCTGTG | CATCCGCCTTTGAATCCATCTCTTG |
| INFγ | ACAGCAAGGCGAAAAAGGATG | TGGTGGACCACTCGGATGA |
| TNF-α | CCCTCACACTCAGATCATCTTCT | GCTACGACGTGGGCTACAG |
| CCL2 | GTGATGGAGGGGGTCAGGA | GGGATGGGACAGCCTAAACT |
| IL-13 | AACGGCAGCATGGTATGGAGTG | TGGGTCCTGTAGATGGCATTGC |
| FN | CCCTATCTCTGATACCGTTGTCC | TGCCGCAACTACTGTGATTCGG |

**Supplemental Table S4. The sequences of the oligonucleotides used for qRT-PCR of the indicated genes.**
